# Radiographic Morphometric Analysis of the Distal Radius in the Tamil Nadu Population: A Retrospective Study

**DOI:** 10.7759/cureus.62226

**Published:** 2024-06-12

**Authors:** Daliparthi Geeta Anasuya, Arun Kumar, Sabari Arasu, Jeevithan Shanmugam, M Vijaianand, Duraisamy Praveen

**Affiliations:** 1 Department of Anatomy, Kovai Medical Centre and Hospital (KMCH) Institute of Health Sciences and Research, Coimbatore, IND; 2 Department of Radiology, Kovai Medical Centre and Hospital (KMCH) Institute of Health Sciences and Research, Coimbatore, IND; 3 Department of Community Medicine, Kovai Medical Centre and Hospital (KMCH) Institute of Health Sciences and Research, Coimbatore, IND; 4 Department of Orthopedics, Kovai Medical Centre and Hospital (KMCH) Institute of Health Sciences and Research, Coimbatore, IND

**Keywords:** retrospective study, tamil nadu population, morphometric parameters, radiographic analysis, distal radius

## Abstract

Background and objectives

Morphometric parameters such as radial inclination, palmar tilt, radial height, and ulnar variance exhibit considerable variations influenced by geographical, ethnic, racial, and individual factors. These parameters are pivotal in the context of distal radius fractures, distal radius plate design, and kinesiology. Understanding these variations is crucial for surgical precision and predicting complications.

Methods

This observational, retrospective study, conducted in a single hospital, aimed to determine the morphometric values of the distal end radius, specifically in the South Indian population. We analyzed 300 plain radiographs, encompassing 53.7% males and 46.3% females, with ages ranging from 17 to 89 years (mean age: 41.05 ± 15.8). Radial inclination, radial height, palmar tilt, and ulnar variance were measured on posteroanterior views, while palmar tilt was assessed on lateral wrist X-rays.

Results

In our study, significant gender-based and side-specific differences were observed. The mean length of the styloid process, palmar tilt, ulnar variance, anteroposterior diameter of the radius, transverse diameter of the radius, oblique width of the radius, and carpal height of the radius exhibited notable variations between males and females. Similarly, significant differences were noted between the right and left sides concerning ulnar variance and teardrop angle. Among males, a significant difference was observed only in the teardrop angle between the right and left sides (59.11 ± 7.25 vs. 62.01 ± 7.97).

Conclusion

The findings underscore the importance of recognizing local morphometric variations in the South Indian population. This knowledge not only enhances the ability to restore normal alignment post-distal radius fractures but also provides fundamental values for future research endeavors within the local demographic. The study acts as a foundational resource for advancing our understanding of the normal anatomy and variations in the distal radius, facilitating improved clinical outcomes and tailored surgical interventions.

## Introduction

Fractures of the distal end radius (DER) constitute 8-15% of all upper limb fractures in adults [1]. The efficacy of DER fracture reduction is primarily evaluated by restoring the pre-fractured values of radial inclination and volar tilt. Altered radial height, radial inclination, and dorsal angulation lead to changes in wrist kinematics.

A comprehensive understanding of normal distal morphometry is important in orthopedic practice [2,3]. Key parameters, including radial height, radial inclination, palmar tilt, and ulnar variance, play a pivotal role in various scenarios such as DER fractures, DER plate design, and kinesiology [4-7].

Notably, no studies have extensively explored the morphometry of the distal radius in the Tamil Nadu population. Therefore, our study aims to determine the radiographic morphometric parameter values of the DER within the Tamil Nadu population.

## Materials and methods

Study design

This is a single-center, retrospective observational study conducted at Kovai Medical Centre and Hospital Institute of Health Sciences and Research.

Ethical consideration

Written informed consent was obtained. Approval for this study was obtained from the Scientific Committee of our institution (approval number 06/IHEC/2023).

Study criteria

The study includes wrist radiographs, either right or left, from patients aged 18 years and above (to ensure that the epiphyseal fusion was complete in both females and males) who sought treatment at the orthopedics department between April 2023 and October 2023. Patients having posteroanterior and lateral radiographs of the wrist with neutral rotation were considered for inclusion. Patients with radiographs that were not centered over the wrist or lacked true views were excluded from the study. Additionally, individuals with open physis, previous injuries and/or surgeries to the DER, or pathological conditions like arthritis were also excluded.

Procedure

Data were collected from outpatient department records and picture archiving and communication system and then entered into an Excel sheet (Microsoft Corporation, Redmond, Washington, United States). We collected data that included patient demographics, radial height, radial inclination, palmar tilt, ulnar variance, anteroposterior diameter, transverse diameter, carpal height radius, and teardrop. All radiographs were taken with standard magnification.

Assessments

**Figure 1:**
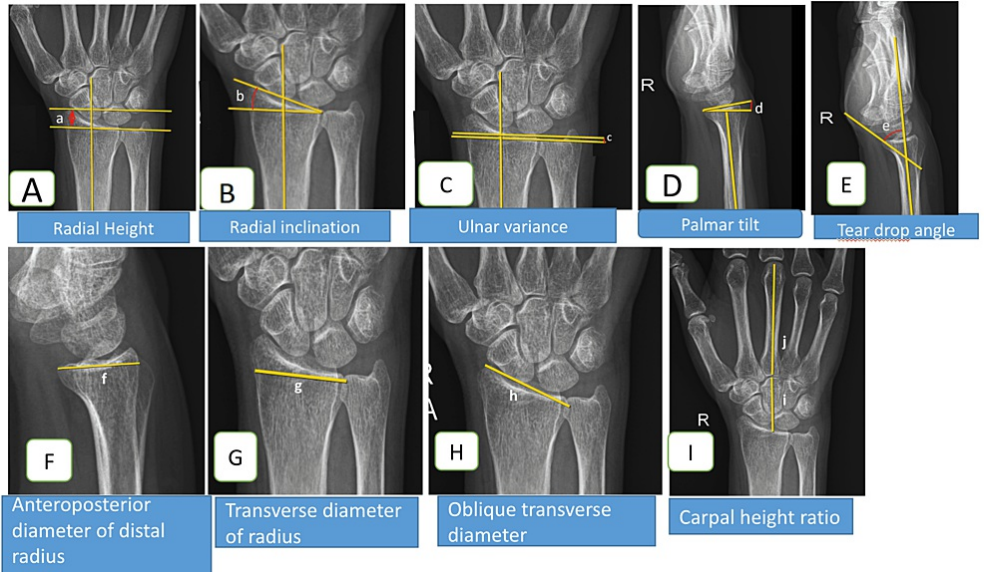
Radiographic measurement techniques for distal radius morphometric variables Figure 1A illustrates the determination of radial height (a), which involves measuring the distance in millimeters between two parallel lines drawn perpendicular to the shaft of the radius. One line is positioned at the distal end of the ulnar head, while the other is located at the radial styloid. Figure 1B demonstrates the measurement technique for radial inclination (b), defined as the angle between a line perpendicular to the radial shaft and a line connecting the distal end of the distal radioulnar joint to the radial styloid. Palmar tilt (c), depicted in Figure 1C, is determined by the angle between a line perpendicular to the radial shaft and a line connecting the volar and dorsal rims of the distal radius. In Figure 1D, ulnar variance (d) is measured as the vertical distance between two lines drawn perpendicular to the long axis of the radius. One line runs parallel to the medial corner of the articular surface of the radius, while the other runs parallel to the most distal aspect of the articular surface of the ulnar head. Figure 1E illustrates the teardrop angle (e), representing the angle between the radial shaft and the central axis of the teardrop, a U-shaped outline of the volar lip of the distal radius. Figure 1F showcases the measurement of the anterior-posterior distance (f), which is the distance between the apices of the dorsal and volar rims of the lunate facet, measured in lateral view. Figures 1G and 1H illustrate the calculation of the transverse diameter (g) and oblique transverse diameter (h), respectively. These measurements represent the greatest width of the distal radius, both in a transverse and oblique direction. Finally, Figure 1I illustrates the carpal height ratio, calculated by dividing the carpal height (i) by the length of the third metacarpal (j).

Radial height is determined by measuring the distance in millimeters between two parallel lines drawn perpendicular to the shaft of the radius. One line is positioned at the distal end of the ulnar head, and the other is located at the radial styloid as shown in Figure 1A. The radial inclination is defined as the angle formed between a line perpendicular to the radial shaft and another line connecting the distal end of the distal radioulnar joint to the radial styloid as shown in Figure 1B. Palmar tilt is determined by the angle between a line perpendicular to the radial shaft and a line connecting the volar and dorsal rims of the distal radius as shown in Figure 1C. Ulnar variance is defined as the vertical distance between two lines drawn perpendicular to the long axis of the radius. One line runs parallel to the medial corner of the articular surface of the radius, while the other runs parallel to the most distal aspect of the articular surface of the ulnar head as shown in Figure 1D [8]. The angle between the radial shaft and the teardrop’s central axis, the U-shaped outline of the volar lip of the distal radius, is known as the teardrop angle as shown in Figure 1E. The distance between the apices of the dorsal and volar rims of the lunate facet is known as the anterior-posterior distance, and it is measured in lateral view as shown in Figure 1F. The greatest width of the distal radius both transversely and obliquely was used to calculate the transverse diameter and oblique transverse diameter as shown in Figure 1G and 1H. The carpal height ratio is calculated by dividing the carpal height by the length of the third metacarpal as shown in Figure 1I. All mentioned radiological measurements were cross-checked by two observers who were part of this study, and the mean value of these observations was taken in preparing the final data table.

Statistical analysis

The data was entered into Microsoft Excel and analyzed using IBM SPSS Statistics for Windows, Version 27.0 (Released 2020; IBM Corp., Armonk, NY, USA). Descriptive statistics were presented as frequency/percentages for discrete variables and mean ± SD for continuous variables. The relationship between the measured parameters concerning gender and side was assessed through an independent sample t-test, while the association with age groups was analyzed using a one-way ANOVA. The significance level for the analysis was set at p < 0.05.

## Results

In the comprehensive analysis of 300 wrist joint plain radiographs, 161 (53.7%) were males, and 139 (46.3%) were females. The mean age of the studied population was 41.05 ± 15.8 years, ranging from 17 to 89 years. Carpal height averaged 0.52 ± 0.04 mm (range: 0.43-0.69 mm), with statistically significant differences noted between males (0.53 ± 0.04) and females (0.51 ± 0.03). Palmar tilt, averaging 9.89º ± 4.02º (1-19º), displayed significant gender differences: 9.29º ± 3.84º in males and 10.58º ± 4.12º in females (p = 0.005). The Ulnar variance distribution showed 133 (44.3%) negative, 72 (24%) positive, and 95 (31.7%) neutral values. The mean ulnar variance was -0.30 ± 1.35 mm, with significant gender variation and pronounced differences among negative, positive, and neutral classifications as shown in Table 1 and Table 2.

**Table 1:** Sociodemographic variables of study participants

Variable	Frequency (n)/percentage (%)
Gender	
Male	161 (53.7%)
Female	139 (46.3%)
Side	
Right	151 (50.3%)
Left	149 (49.7%)
Age group (years)	
18-30	100 (33.3%)
31-45	80 (26.7%)
46-60	70 (23.3%)
61-75	50 (16.7%)

**Table 2:** Distribution of various parameters of the distal end of radius * indicates a statistically significant p-value (p < 0.05).

Parameter	Mean ± SD	Range (Min-Max)	p-value
Age (years)	41.05 ± 15.80	17-89	0.045
Inclination angle (º)	25.09 ± 3.09	16-35	0.001*
Length of styloid process (mm)	10.97 ± 1.60	7.0-16.0	0.003*
Palmar tilt (º)	9.89 ± 4.02	1-19	0.003*
Ulnar variance (mm)	-0.30 ± 1.35	-5.3-5.0	0.005*
Anteroposterior diameter (mm)	23.22 ± 2.63	16.3-32.0	0.003*
Transverse diameter of radius (mm)	29.04 ± 2.87	23.0-38.4	0.005*
Oblique width of radius (mm)	31.12 ± 2.90	22.9-41.2	0.003*
Carpal height ratio	0.52 ± 0.04	0.43-0.69	0.004*
Teardrop angle (º)	61.08 ± 7.72	41-77	0.005*

Radial inclination, measuring 25.09º ± 3.09º, showed no significant gender disparity (males: 24.99º ± 3.25º, females: 25.21º ± 2.91º, p = 0.5). The mean styloid process length was 10.97 ± 1.60 mm, with significant gender differences: 11.51 ± 1.57 mm in males and 10.34 ± 1.38 mm in females. The anteroposterior diameter of the DER (23.22 ± 2.63 mm) exhibited significant gender differences (males: 24.52 ± 2.24 mm, females: 21.71 ± 2.22 mm). Similarly, the transverse diameter (29.04 ± 2.87 mm) varied significantly between males (30.81 ± 2.37 mm) and females (26.99 ± 1.87 mm). The oblique width of the radius also displayed a significant difference between males (32.77 ± 2.54 mm) and females (29.21 ± 1.97 mm) (Table 3).

**Table 3:** Summary of the measured parameters and their differences between males and females, analyzed using an independent sample t-test p < 0.005: statistically significant

Parameter	Males (mean ± SD)	Females (mean ± SD)	p-value
Radial height (mm)	11.51 ± 1.57	10.34 ± 1.38	<0.001
Palmar tilt (º)	9.29 ± 3.84	10.58 ± 4.12	0.005
Ulnar variance (mm)	-0.30 ± 1.35	-0.15 ± 1.32	0.05
Radial inclination (º)	24.99 ± 3.25	25.21 ± 2.91	0.5
Styloid process length (mm)	11.51 ± 1.57	10.34 ± 1.38	<0.001
AP diameter (mm)	24.52 ± 2.24	21.71 ± 2.22	<0.001
Transverse diameter (mm)	30.81 ± 2.37	26.99 ± 1.87	<0.001
Oblique width (mm)	32.77 ± 2.54	29.21 ± 1.97	<0.001
Carpal height (mm)	0.53 ± 0.04	0.51 ± 0.03	0.01

Regarding laterality, significant differences were observed in teardrop angle between the right (59.11º ± 7.25º) and left (62.01º ± 7.97º) sides among males. For females, the measured parameters showed no significant differences between the right and left sides, except for the length of the styloid (10.10 ± 1.32 mm vs. 10.58 ± 1.42 mm) and ulnar variance (-0.37 ± 1.30 mm vs. 0.16 ± 1.32 mm) (Table 4, Table 5).

**Table 4:** Differences in measured parameters between the right and left sides, analyzed using an independent sample t-test p < 0.005: statistically significant

Parameter	Right side (mean ± SD)	Left side (mean ± SD)	p-value
Radial height (mm)	11.00 ± 1.60	10.94 ± 1.58	0.5
Palmar tilt (º)	9.89 ± 4.02	9.89 ± 4.02	1
Ulnar variance (mm)	-0.30 ± 1.35	-0.30 ± 1.35	1.0
Radial inclination (º)	25.09 ± 3.09	25.09 ± 3.09	1
Teardrop angle (º)	59.11 ± 7.25	62.01 ± 7.97	0.05
Styloid process length (mm)	10.34 ± 1.38	10.34 ± 1.38	1
AP diameter (mm)	23.22 ± 2.63	23.22 ± 2.63	1
Transverse diameter (mm)	29.04 ± 2.87	29.04 ± 2.87	1
Oblique width (mm)	32.77 ± 2.54	32.77 ± 2.54	1

**Table 5:** Comparison of measured parameters across different age groups using one-way ANOVA p < 0.005: statistically significant

Parameter	18-30 years (mean ± SD)	31-45 years (mean ± SD)	46-60 years (mean ± SD)	61-75 years (mean ± SD)	p-value
Radial height (mm)	11.50 ± 1.57	11.30 ± 1.50	11.00 ± 1.48	10.50 ± 1.40	0.01
Palmar tilt (º)	10.50 ± 4.00	10.00 ± 3.90	9.50 ± 3.80	9.00 ± 3.70	0.05
Ulnar variance (mm)	-0.10 ± 1.30	-0.20 ± 1.35	-0.30 ± 1.40	-0.40 ± 1.45	0.05
Radial inclination (º)	25.50 ± 3.20	25.30 ± 3.15	25.10 ± 3.10	24.90 ± 3.05	0.1
Teardrop angle (º)	60.50 ± 7.50	60.00 ± 7.40	59.50 ± 7.30	59.00 ± 7.20	0.1
Styloid process length (mm)	11.50 ± 1.57	11.30 ± 1.50	11.00 ± 1.48	10.50 ± 1.40	0.01
AP diameter (mm)	24.50 ± 2.25	24.00 ± 2.20	23.50 ± 2.15	23.00 ± 2.10	0.05
Transverse diameter (mm)	30.50 ± 2.35	30.00 ± 2.30	29.50 ± 2.25	29.00 ± 2.20	0.05
Oblique width (mm)	32.50 ± 2.55	32.00 ± 2.50	31.50 ± 2.45	31.00 ± 2.40	0.05

## Discussion

The evaluation of distal radius fracture reduction hinges on achieving anatomical precision in morphometric measurements. Orthopedic surgeons often turn to the established standard values of Gartland and Westley [9] as a reference. However, it is important to recognize that morphometric parameters can vary based on factors such as country, race, ethnic background, and the individual physique of the patient. In our current study, we aim to contribute valuable insights by presenting data specific to the local population in Tamil Nadu. This approach ensures a more nuanced understanding of fracture reduction considerations tailored to the unique characteristics of this demographic.

In our study, the mean radial height was observed to be 10.97 ± 1.60 mm (range: 7-16 mm). In comparison, the Orthopaedic Trauma Association’s (OTA) reference [9] value falls between 11 and 13 mm. Notably, Hadi and Wijiono [7] found a median radial height of 11.51 ± 1.6mm in the Indonesian population, with Nekkanti et al. [10] also reporting significance in this parameter as shown in Table 6.

**Table 6:** Comparison of our study to Western literature N = sample size

Morphometric parameters of the distal radius	Chan et al. [4] (Indian, N = 21)	Chan et al. [4] (Malaysian, N = 38)	Chan et al. [4] (Chinese, N = 12)	Hadi and Wijiono [7] (Indonesian, N = 400)	Bilgin et al. [21] (Turkish, N = 981)
Radial height (mm)	Not observed	Not reported	Not reported	11.36 ± 1.66	13.6 ± 2.1
Radial inclination (º)	27 ± 3.18	24.8 ± 3.03	24.1 ± 3.77	Not reported	26.7 ± 3.3
Palmar tilt (º)	13.0 ± 3.57	12.9 ± 3.78	11.8 ± 2.77	Not reported	15.4 ± 4.3
Ulnar variance (mm)	0.13 ± 0.70	Not reported	Not reported	Not reported	0.8 ± 1.9

These variations suggest a potential tendency among treating doctors to over-distract the distal radius during procedures such as plating, possibly driven by the desire to meet standard radiographic criteria. This over-distraction of the distal radius could result in excessive strain on radiocarpal ligaments, subsequently impacting the functional outcome of these injuries in terms of hand grip and the range of movements in the distal radius. Careful consideration and adjustment of procedural techniques may be warranted to optimize both radiographic outcomes and functional results in the management of distal radius fractures.

In our study, radial inclination was measured at 25.09º ± 3.09º (range: 16-35º). This finding deviates slightly from the OTA standard reference value, which suggests a range of 23º (range: 13-30º) [9]. Interestingly, Chan et al. [4] reported a similar radial inclination of 25.1 ± 3.42 in their study, while Nekkanti et al. [10] found a lower value of 21.58º ± 3.35º. Additionally, Gupta et al. [11], in their 2015 cadaveric study, observed radial inclination ranging from 24º to 25.6º. These variations in radial inclination across studies underscore the importance of considering population-specific factors and methodological differences in the interpretation of radiographic measurements.

In our study, the palmar tilt averaged 9.89º ± 4.02º (range: 1-19º), aligning well with the OTA reference range of 1-21º. Comparatively, Chan et al. [4] found a palmar tilt of 12.6º ± 3.55º in the Malaysian population. Nekkanti et al. [10] reported a slightly narrower range in their study, with palmar tilt measured between 10.92º ± 2.86º and 11.62º ± 3.36º, as shown in Table 7. These variations imply that the anatomical angles vary among different sets of populations, and it is important to know these differences while restoring the normal anatomy at the time of fracture fixation. The potential reasons for these differences encompass genetic and ethnic variations, lifestyle factors, as well as sample size and demographics.

**Table 7:** Comparison with other Indian studies and reference values N = sample size

Morphometric parameters of the distal radius	Pritishkumar et al. [22] (Indian, N = 420)	Vardhan et al. [20] (Jharkhand, N = 120)	Agarwala and Vetri [23] (Southern Assam, N = 200)	Nekkanti et al. [10] (N = 310)	Maheswaran et al. [24] (South Indian population, N = 100)	Tornetta et al. [8] (N = 320)	Present study (Tamil Nadu population, N = 300)
Radial height (mm)	11.31 ± 4.9	11.33 ± 4.7	1.03 ± 0.21	0.88 ± 0.26	11.30 ± 0.30	11-12 (8-18)	10.97 ± 1.60
Radial inclination (º)	23.27 ± 7.42	23.29 ± 7.4	21.85 ± 2.76	21.58 ± 3.35	22.54 ± 2.18	22-23 (12-23)	25.09 ± 3.09
Palmar tilt (º)	10.07 ± 5.28	10.09 ± 5.23	11.99 ± 2.88	11.36 ± 3.16	12.01 ± 1.88	11-22 (0-28)	9.89 ± 4.02
Ulnar variance (mm)	0.66 ± 2.46	Not reported	0.39 ± 1.43	Not reported	0.44 ± 1.03	Neutral	-0.30 ± 1.35

In our study, the mean ulnar variance was found to be negative in both males and females. A positive ulnar variance was observed in 72 patients, while 95 patients exhibited a neutral ulnar variance. The OTA criteria consider neutral ulnar variance as the standard reference value. Comparing our findings to other studies, Nekkanti et al. [10] found a negative ulnar variance in 108 patients (34.8%). In the Indian population, Chan et al. [4] and Mishra et al. [12] observed positive ulnar variance of 0.13 ± 0.72 mm and 0.66 ± 2.46 mm. Conversely, cadaveric studies by Schuind et al. [13], Werner et al. [14], and Altissimi et al. [15] reported negative ulnar variance.

Chan et al. [4] observed statistically significant variations in ulnar variance among Chinese and Malaysian populations, and studies have indicated that ulnar variance values may alter in relation to age and gender [16]. A negative ulnar variance can potentially lead to avascular necrosis of the lunatum due to increased loading on the radius-lunatum-capitatum-third metacarpal bone [17]. Gelberman et al. [18] reported a higher susceptibility to Kienböck’s disease with negative ulnar variance in whites. These diverse observations underscore the multifactorial nature of ulnar variance and its clinical implications.

Hadi and Wijiono [7] noted statistical significance in radial height, radial inclination, palmar tilt, and ulnar variance between males and females within the Indonesian population. The functional outcome assessment for distal radius fracture treatment was introduced by Gartland and Werley in 1951 [9]. While numerous studies did not find significant statistical differences in morphometric parameters between the right and left wrists, there is limited literature discussing gender-specific distributions of these parameters. In our study, a noteworthy gender-based disparity was observed in radial height, palmar tilt, ulnar variance, length of the styloid process, anteroposterior diameter, transverse diameter, and oblique width of the radius. Nekkanti et al. [10] and Mishra et al. [12] reported a statistical difference between male and female populations, specifically in radial height. In a study on the Nepalese population by Kadel et al. [19], similar parameters were measured using dry radii, with mean palmar tilt and radial inclination at 9.72º and 23.62º, respectively. However, the values of other parameters were found to be lower compared to our present study [20-24], as shown in Table 8. The observed gender-based disparities and differences in morphometric parameters have significant implications. Understanding these variations allows for personalized treatment approaches and the establishment of gender-specific reference standards, improving functional outcomes. The potential reasons for these differences include genetic and ethnic variations, hormonal influences, sample size and demographic differences, and lifestyle factors.

**Table 8:** Comparison with other Indian studies and reference values N = sample size

Morphometric parameters of distal radius	Gartland and Werley [9] (N = 425)	Altissimi et al. [15] (N = 320)	Schuind et al. [13] (N = 120)	Werner et al. [14] (N = 58)	Nakamura et al [16] (N = 325)
Radial height (mm)	Not reported	Not reported	Not reported	-0.1 ± 1.4	Not reported
Radial inclination (º)	23 (13-30)	16-28	24 (19-29)	30	Not reported
Palmar tilt (mm)	11 (1-21)	0-18	Not reported	6	Not reported
Ulnar variance (mm)	Not reported	-2.5 to + 3.1	-4.2 to 2.3	-0.1 ± 1.4	0.20 ± 1.39

The study’s primary limitation lies in its retrospective nature, which may introduce potential biases and limitations in data collection. Additionally, the reliance on radiographic data may not fully capture the nuanced clinical context, limiting the interpretation of morphometric parameters. Lastly, the study’s scope is specific to the Tamil Nadu population, potentially restricting the generalizability of the findings to broader demographic groups.

## Conclusions

This study underscores the significance of achieving optimal alignment in distal radius fractures within the Tamil Nadu population. A nuanced understanding of the variations in these morphometric parameters specific to the local demographic empowers surgeons to enhance surgical precision, anticipate potential complications such as diminished grip strength and mid-carpal instabilities, and avoid issues associated with over-distracting procedures. This knowledge contributes to the refinement of surgical approaches and ultimately improves patient outcomes in the context of distal radius fractures. Further prospective investigations are warranted to comprehensively understand and address the intricate interplay of gender-specific factors in wrist morphology and related clinical implications.
